# Single‐Molecule Investigation of Load‐Dependent Actomyosin Dissociation Kinetics for Cardiac and Slow Skeletal Myosin

**DOI:** 10.1002/smll.202406865

**Published:** 2024-10-07

**Authors:** Tianbang Wang, Arnab Nayak, Theresia Kraft, Mamta Amrute‐Nayak

**Affiliations:** ^1^ Institute of Molecular and Cell Physiology Hannover Medical School 30625 Hannover Germany

**Keywords:** actomyosin, cardiac ventricular myosin, mechanosensitivity, optical trapping, single‐molecule studies

## Abstract

Myosins are ATP‐powered, force‐generating motor proteins involved in cardiac and muscle contraction. The external load experienced by the myosins modulates and coordinates their function in vivo. Here, this study investigates the tension‐sensing mechanisms of rabbit native β‐cardiac myosin (βM‐II) and slow skeletal myosins (SolM‐II) that perform in different physiological settings. Using mobile optical tweezers with a square wave‐scanning mode, a range of external assisting and resisting loads from 0 to 15 pN is exerted on single myosin molecules as they interact with the actin filament. Influenced of load on specific strongly‐bound states in the cross‐bridge cycle is examined by adjusting the [ATP]. The results implies that the detachment kinetics of actomyosin ADP.Pi strongly‐bound force‐generating state are load sensitive. Low assisting load accelerates, while the resisting load hinders the actomyosin detachment, presumably, by slowing both the Pi and ADP release. However, under both high assisting and resisting load, the rate of actomyosin dissociation decelerates. The transition from actomyosin ADP.Pi to ADP state appears to occur with a higher probability for βM‐II than SolM‐II. This study interpret that dissociation of at least three strongly‐bound actomyosin states are load‐sensitive and may contribute to functional diversity among different myosins.

## Graphical abstract

Inherent sensitivity toward external load forms a basis for the myosin motors physiological adaptation. Here, this study shows that the mechanical performance of cardiac and slow skeletal myosin II is determined by the lifetime of force‐producing actin bound myosin states. Load modulates the bound duration, and the steady‐state abundance of actin‐bound and detached myosin intermediates, having direct implications on physiological function.

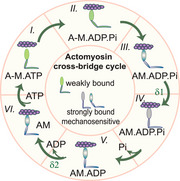

## 1 Introduction

Cardiac and skeletal muscle can bear load at a constant length, i.e., under isometric conditions, or undergo rapid shortening under low load conditions. The muscle's shortening velocity decreases with increasing load. Accordingly, muscle performance is determined by the force‐velocity relationship in vivo. The load dependence of the muscle function was predicted In the classical mathematical model of AF Huxley.^[^ ^1^ ^]^ Rate of force development and sensitivity to load depends on the fiber types, i.e., slow twitch‐type 1 and fast‐twitch type 2 fibers in muscles.^[^ ^2^ ^]^ The fiber functions, categorized by their enzymatic properties and maximum shortening velocity, are determined by the specific myosin isoforms, namely fast and slow myosin II expressed in these muscles.^[^ ^3^, ^4^ ^]^ Similarly, ventricular and atrial myosin isoforms confer distinct force‐producing and force‐bearing features in the heart muscle. With the advance of single‐molecule experiments, it has been possible to directly demonstrate the effect of load on the actin‐myosin dissociation kinetics for different myosin isoforms. Duration of actomyosin bound state prolonged or shortened with increasing load can be distinguished as catch‐bond or slip‐bond behavior, respectively.^[^ ^5^ ^]^

Almost all known myosins utilize the same biochemical pathways employing the energy from ATP hydrolysis for mechanical activity, i.e., generating force to displace or to move along the actin filaments, or to maintain the tension through prolonged actomyosin associations.^[^ ^6^ ^]^ In this well‐characterized chemomechanical cycle, upon ATP binding, myosin dissociates from actin. ATP is hydrolyzed to ADP and Pi and myosin resumes the pre‐powerstroke configuration as the lever arm is reprimed. In the ADP.Pi state, myosin associates weakly and then strongly with actin filament. Pi release is closely coupled with the generation of the first powerstroke, while subsequent ADP release from the active site results in the second powerstroke. Following ADP release from the active site, myosin adopts rigor conformation with actin. The cycle continues with a new ATP molecule binding to the myosin and dissociation of the actomyosin complex.^[^ ^7^, ^8^, ^9^ ^]^ Within a chemomechanical cycle, lifetimes of the force‐generating states are of particular interest as they determine the muscle shortening or contraction speed. Importantly, external load can modulate biochemical and mechanical transition rates and equilibrium constants. The affected transition states and the extent of influence on different states can determine the motor's physiological function to become either a load‐sensor, tunable processive, or a non‐processive motor.^[^ ^10^ ^]^ To understand the mechanosensitive nature of specific motors, the weak binding states, the transition from weak to strong actomyosin complex formation, strongly bound force‐generating, and rigor states have been the subject of intense investigations. As the motors experience a range of forces in a cell, gaining insight in their individual and collective response under tension is of high importance to understand the fundamental mechanisms underlying the respective myosins role. Besides, alterations in the rate or equilibrium constants of specific biochemical transition states and in the mechanical parameters under various stress conditions can determine the progression and the severity of diseases in muscle and cardiac pathologies.

Direct measurements using single molecule measurement tools have been instrumental in demonstrating the load‐affected transition states in actomyosin ATPase cycle. The rate of ADP release that limits actin detachment and thereby motile speed has been found to be sensitive to external load in multiple myosin isoforms, including processive motors myosin V^[^ ^11^ ^]^ and myosin VI,^[^ ^12^ ^]^ low‐duty ratio motors such as smooth muscle myosin II,^[^ ^13^ ^]^ β cardiac myosin II,^[^ ^14^, ^15^ ^]^ and myosin 1.^[^ ^16^, ^17^ ^]^ For the myosin 1c isoform, the ATP‐induced actomyosin dissociation step was found to be sensitive to an external load, suggesting that even different isoforms of the same motor can react differently to applied force.^[^ ^18^ ^]^ Myosin VI also showed a load dependence of ATP association rate.^[^ ^12^ ^]^

Conventional skeletal muscle myosin II is a holoenzyme composed of myosin heavy (MyHC) and light chains (MLC). *MYH7* gene product β‐myosin heavy chain (β‐MyHC) is expressed in ventricular myocardium and in slow‐twitch skeletal muscle, Musculus soleus (M. soleus). Due to their identical heavy chain composition, the myosins expressed in these tissues were considered functionally equivalent. However, we recently demonstrated that the actin gliding speed differs by threefold, with cardiac myosin being faster than the SolM‐II.^[^ ^19^, ^20^ ^]^ Consistent with these observations, single‐molecule analysis further revealed a higher actomyosin detachment rate for ventricular myosins. The sole difference in the motor composition, i.e., the presence of MLC1 (MLC1sa) with 13 additional amino acids in SolM‐II, raised a possibility that 50% of myosin heads bearing this light chain (MLC1sa) are sufficient to cause the functional difference in the two motor complexes. As the two myosins fulfill distinct physiological requirements, i.e., regular cycling during cardiac contraction versus voluntary contraction in slow muscles, they feasibly perform under varying external loads. Therefore, we set out to investigate the details of the responsible force‐sensing states in the chemomechanical cycle for these myosins.

Using single‐molecule optical trapping with an advanced setup, we identified previously undetected strongly bound actomyosin states that respond to load by altering the detachment kinetics. Surprisingly, both resistive and assistive load decelerated the actomyosin detachment rates in SolM‐II as well as βM‐II. In accordance with earlier observations, the ADP bound state showed actomyosin catch bond behavior, i.e., the increase of actomyosin bound duration (faster dissociation) under increasing load. Although the resisting force‐dependent slowing down of actomyosin dissociation was reported earlier, the same effect on increasing assisting load was never described. Furthermore, based on our observations, the transition from the ADP.Pi to ADP state appeared to be faster for the βM‐II. Ventricular βM‐II showed load‐dependent changes in the detachment kinetics in ADP.Pi states that were distinct from SolM‐II, suggesting the myosin's functional adaptation to fulfill their specific task.

## 2 Results

### 2.1 Actomyosin Detachment Kinetics for M. soleus myosin II are Load‐Dependent

**Figure 1:**
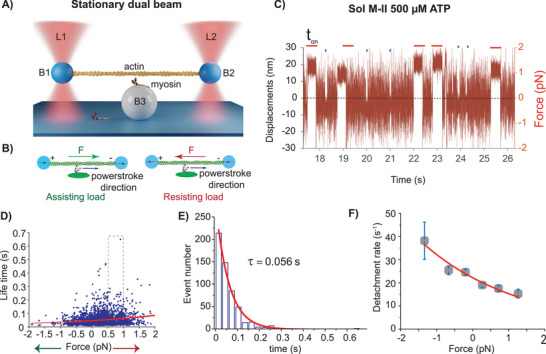
A) Stationary dual beam set up. The actin filament is held between the two beads (blue) B1 and B2 trapped with focused laser beams L1 and L2. The laser beam position remains fixed. Myosin is adsorbed onto the pedestal bead (white). Illustrated elements are not to scale. B) Actomyosin interaction when the dumbbell exerts external force (F) in the same or opposite direction to that of the powerstroke direction. “+” and ‘−’ signs indicate the actin polarity and represent the plus and minus end of an actin filament. C) Typical bead position time record elucidating the dumbbell undergoing a Brownian motion shown as large amplitude fluctuations. The intermittent interaction between actin and myosin impedes the free dumbbell movement, thus reducing the fluctuation amplitude as indicated in the record with red horizontal lines (longer‐duration events) or blue points (shorter‐duration events). Displacement (nm) and trap stiffness (pN nm^−1^) are used to estimate the force shown on the right Y‐axis. D) Scatter plot showing individual actomyosin interaction lifetime events at different forces measured at 500 µm ATP. The plot was fitted with a single exponential Arrhenius equation (red line, Maximum likelihood estimation (MLE)) that allows evaluation of the force‐dependent lifetime or detachment rate of the actomyosin complex. The green arrow along the x‐axis indicates increasing assistive load, and the red arrow‐ increasing resistive load. E) Histogram of lifetimes of the binding events. An example showing the events shown in figure D (in dotted rectangle) between the force range of 0.5–1 pN are binned, n = 541. MLE was employed to calculate the average lifetime (τ) and thereby detachment rate (1/τ) of 17.55 ± 1.37 s^−1^. F) Detachment rates over force plot. Events in panel D were binned using 0.5 pN binwidth to pool the events within corresponding force range. For the last data points, the events in 1–2 pN force range were pooled, as fewer events were detectible in this range. Plot fitted with user defined single exponential function (Arrhenius equation): *k* = *ko* exp (‐F*d/*k_B_* *T) in origin lab tool, yielded *k_0_* of 22.18 ± 0.88 s^−1^ and d of 1.53 ± 0.17 nm. N = 25, n = 2578. N‐ number of myosin molecules, n‐ number of binding events.

Using single‐molecule optical trap measurements, the lifetimes of binding events between a native myosin motor and actin under various loads can be directly measured at specific ATP concentrations. We probed the influence of external load on actomyosin dissociation kinetics at low and high ATP concentrations. At low [ATP], the actomyosin dissociation is limited by the ATP binding to the rigor complex, allowing the properties of the rigor state to be analyzed. High ATP concentrations were chosen to determine the ADP dissociation under a range of loads as individual molecules of SolM‐II interact with actin. In a 3‐bead assay (illustrated in **Figure** **1**A) as the dumbbell undergoes Brownian motion across the myosin‐hosting bead, the myosin can bind to actin. Typically, during the actomyosin crossbridge cycle, as a plus‐end directed motor, myosin associates toward the F‐actin barbed‐ or plus‐end and generates a powerstroke in the direction of the pointed‐ or minus‐end of actin (cf. Figure 1B). When the trapping beam applies load in the same direction as the powerstroke, the myosin head senses assisting load, whereas resisting load is experienced by the myosin when the trap moves in the opposite direction to that of the powerstroke direction. Thus, the dumbbell movement in the same direction as a powerstroke or in the opposite direction causes the myosin head to experience a varying range of assisting or resisting force, respectively. A characteristic displacement over time record shows intermittent actomyosin interactions for a single myosin molecule (Figure 1C), with blue dots and red horizontal lines representing a duration of individual binding events “t_on_”. Additional original data traces and variance plots for binding event detection are provided in Figure S1 (Supporting Information). The force “F” for the corresponding event is estimated from the displacement from average zero position of bead fluctuations and the trap stiffness. In Figure 1C actomyosin interaction events with varied durations become apparent. In this typical example, the events on the indicated positive displacement side are longer than on the negative displacement side, implying load dependence of the actomyosin bound lifetime. We reasoned that for SolM‐II, the actomyosin interactions at 500 µm ATP primarily represent duration of ADP bound states. Figure 1D showing a scatter plot suggests that increasing the resisting load delays the ADP dissociation rate, i.e., the lifetimes increase while the increasing assisting load results in faster ADP release, as evident from reduced lifetimes (t_on_), respectively. To determine the load (F) dependence of the detachment rate (*k_det_*), the lifetime plot was fitted with an approximation of Arrhenius equation, *k_detach_* = *k_0_* exp [‐F.d/*k_B_*.*T*] using MEMLET tool. From this fit, the detachment rate at zero load or absence of load (*k*_0_) and the distance (d) to the transition state of the rate‐limiting step in the bound state were estimated. “d” is a measure of the myosin's sensitivity to load. A “d” value closer to or higher than the second stroke size signifies higher load dependence, *k_B_* is the Boltzmann constant and T is the absolute temperature in Kelvin. *k*_0_ of 26.98 ± 1.37 s^−1^ and d of 1.53 ± 0.31 nm was estimated for SolM‐II at 500 µm ATP. Alternatively, *k*_0_ and d were estimated by binning the lifetime events as shown in Figure E and F. Figure 1E shows a representative histogram fitted with a single exponential function to derive the average time constant (τ). The detachment rates (1/τ) obtained from such plots for different load range is shown in Figure 1F. The rates fitted with a single exponential function yielded the comparable values for “*k*_0_” (22.18 ± 0.88 s^−1^) and “d” (1.53 ± 0.17 nm) as for scattered plot in Figure 1D.

Earlier we have estimated overall stroke size of ≈6 nm, that comprises the first powerstroke (δ1) of 4.37 nm and second powerstroke (δ2) of 1.74 nm for SolM‐II.^[^ ^21^ ^]^ Overall, SolM‐II displayed a load‐dependent change in cross‐bridge kinetics.

### 2.2 Measurement of Actomyosin Detachment Kinetics Using Square Wave Scanning Approach

**Figure 2:**
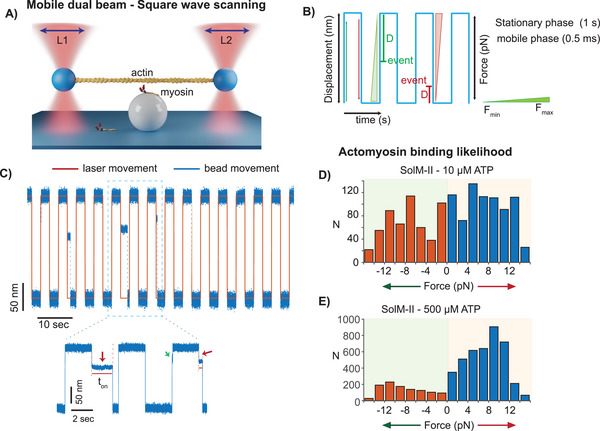
Square wave scanning method. A) The laser beams (L1 and L2) were synchronized to switch between rapid mobile and long stationary phases. The dumbbell thus alternates the direction along the actin filament axis (X‐direction), i.e., sweeps back and forth parallel to an actin filament past the myosin on the platform bead. B) The blue square‐shaped form shows the displacement over time trace when the trapped beads alternate between the mobile and stationary phases as they follow the laser motion. The mobile phase lasts for 0.5 ms while the stationary phase was set between 0.5 and 5 sec as per the experimental requirement. Myosin can bind to actin during the mobile phase and stationary phase. The binding events during the mobile phase are analyzed for load dependence. The actomyosin binding events are indicated during the upward (green) or downward (red) movement. ‘D’ is the distance between mean bead and the laser position, which is used to estimate the external force. F_min_ and F_max_ are minimum and maximum force experienced by the actomyosin crossbridge depending on the binding position during the 180 nm excursion. C) Original data trace showing the bead motion (blue) and the actomyosin interaction events at 500 µm ATP for SolM‐II. The laser motion is shown in red. The record is collected at 500 µm ATP, 0.2 HZ laser frequency, with a 2.5 sec pause and rapid dumbbell movement in a range of 0.5 ms, and excursion of 180 nm. The actomyosin binding events are further highlighted in inset. Events occurring in the different directions are indicated with red and green arrow to indicate the opposing forces experienced by the myosin during its binding to the actin filament. D,E) Actomyosin binding likelihood of SolM‐II to actin filament under the assisting (red bars) and resisting (blue bars) load measured at 10 and 500 µm ATP. 0 denotes the stationary phase of the beads. At 10 µm ATP, the total number of detected events is 776 on the resisting side and 546 on the assisting side yielding a ratio of 1.4. At 500 µm ATP, a total of 4023 and 1089 events were detected under resisting and assisting force, respectively, yielding a ratio of 3.69. Light green and red background indicate the events under the assistive and resistive load, respectively.

The actomyosin detachment kinetics at a relatively lower load range of ± 2 pN were measurable with the data records obtained from a stationary dual‐beam setup (Figure 1D), albeit with lower time resolution (events detection >5 ms). An advanced experimental setup was necessary to examine the influence of higher loads and improve the temporal resolution. We employed a mobile dual‐beam optical trap setup, whereby the square waveform was applied to the dumbbell (**Figure** **2**A). A single bead assay with square wave scanning was first introduced by the Yanagida lab to examine short‐lived weak interactions or the initial contact information between actin and myosin VI.^[^ ^22^ ^]^ In the current study for non‐processive motor myosin II, we employed a 3‐bead assay. The position of the two trapping laser beams and, in turn, optically trapped beads holding the actin filament was controlled by two synchronized electro‐optical deflectors (EODs). Before starting the measurements with the square wave scanning approach, actin polarity was established by recording the actomyosin interactions in the stationary trap routine, and the direction of the power stroke was determined (cf suppl. Figure S2, Supporting Information). In this case, an interaction event is detected based on a reduction in Brownian fluctuations (as described in the earlier section). The resisting (positive) and assisting (negative) forces were defined as the force in the opposite direction and in the direction of the stroke, respectively, as described in Figure 1B. Subsequently, the dual‐beam holding a pre‐stretched actin filament, i.e., dumbbell, was moved rapidly back and forth across the myosin motor anchored on the platform bead, allowing ±180 nm harmonized oscillation of laser beams with a constant speed as illustrated in Figure 2B. During the mobile phase, the dumbbell movement can be restricted when myosin binds to actin. While the laser trap moves on to the next designated position, the dumbbell lags behind until the myosin dissociates from actin. The actomyosin binding further decouples the two beads (Figure S3, Supporting Information). As the beads are drawn toward the center of the laser trap, the leading bead and, thereby, attached actomyosin cross‐bridge experience a definite amount of load. Thus, the distance of the beads to the stationary position of the laser (i.e., trap – bead separation) is a measure for the load experienced by the actin bound myosin. Following actomyosin dissociation, the beads can regain their positions in the trap. Figure 2C shows an example trace with interaction events during 180 nm excursion in alternating directions. Additional original data traces and information on event detection are provided in Figure S4 (Supporting Information) and Experimental Section. During the fast mobile phase of the dumbbell, myosin can bind to actin at different positions across the 180 nm excursion; thereby, the associated motor head experiences varying loads based on the binding positions. The distance between the bead and the next stationary laser position is used to derive the forward pushing (assistive) or backward holding (resistive) load (Figure 2B, the red and green line represents laser movement trajectory). The force was calculated from the difference between the bead and trap position (as shown in Figure 2B,G), *F* = *ΔD*∗*k_L,_* F: load, ΔD: distance between bead position (events) and stationary trap position in the square wave scanning, *k_L_*: trap stiffness. The constant load on the actomyosin cross‐bridge is maintained until the motor releases from the actin filament, and the bead follows the trap movements until the initiation of the next binding event. The dumbbell was moved with frequencies of 0.1–1 Hz. The measurements were performed at low and high ATP concentrations. The low frequency of 0.1 Hz was used to allow for possible binding events with a longer lifetime to be accommodated, for example, at [ATP] of 10 µm. Note that we analyzed interaction events that occurred during the mobile phase of the trap.

### 2.3 Binding Likelihood of Myosin Along the Actin Filament

First, we examined whether there is bias in the initial association of myosin to actin along the long axis of the actin filament as it oscillates in the same or opposite direction of the powerstroke, i.e., minus or toward the plus end of the actin filament. The event location along the actin filament indicates the site of transition of myosin from weak to strongly‐bound actin state. The direction of movement corresponds to the assisting and resisting load experienced by the actomyosin complex when the transition occurs. The association events were evaluated at low ATP concentrations to test if the binding likelihood is affected when the actin filament is moved back and forth across the myosin. Low ATP concentration allowed all the binding events to be observed as the durations of the bound states are longer. The contact points were observed throughout the stretch of the actin filament presented as binding events over force as shown in Figure 2D. The binding likelihood along the actin filament was symmetric, with nearly similar number of interaction events observed under assisting and resisting load.

At higher [ATP] of 500 µm, however, the detectible binding event population was shifted to one side, i.e., under resisting load (Figure 2E), implying that the lifetimes of some events under low assisting load were too short to be detected, confirming the load dependence of the bound durations and thereby the actomyosin detachment kinetics. Interestingly, when the events under an assisting load are compared, the number of events increased under a higher assisting load, e.g., ≈10–15 pN, as compared to the lower assisting load of 4 pN.

### 2.4 Actomyosin Interaction Lifetimes for SolM‐II at Higher Load

**Figure 3:**
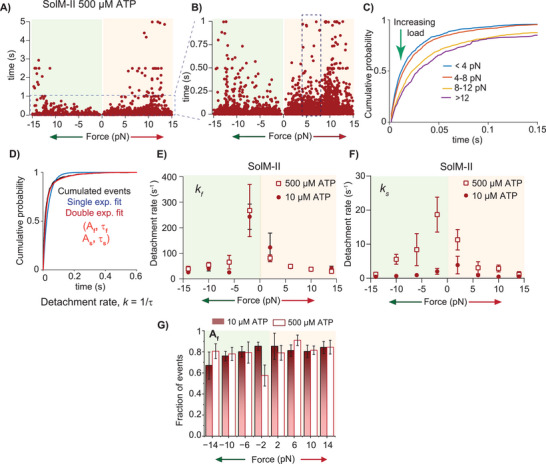
Actomyosin interaction lifetime. A) Duration of actomyosin interaction over external force measured for SolM‐II at 500 µm ATP using a square wave scanning approach. Red dots indicate individual data points. Event lifetimes under assisting and resisting load are shown with a light green and red background, respectively. B) Events shorter than 1 s (dashed box in A) are further expanded to highlight the events with the increased lifetimes under high loads. Total events n = 5112 from 54 individual myosin molecules. C) Cumulative frequency distribution of the binding events are plotted over lifetimes at different resisting loads as indicated. D) The cumulative distribution for binding events pooled from 4‐pN binwidth (i.e., 4 −7.99 pN) fitted with a double exponential function indicated as red fit, n = 1257. Double exponential fit to the distribution yields two populations of events with amplitudes A_f_ and A_s_ with corresponding time constants τ_f_ and τ_s_, respectively. E,F) Fast and slow detachment rates, *k_f_* and *k_s_* derived at 10 and 500 µm ATP are compared for their response to load. *k_f_* and *k*_s_ were estimated from a reciprocal of time constants τ_f_ and τ_s_, for different force range as described in panel D. G) The fraction of events (A_f_) corresponding to the first population (i.e., *k_f_)* at various loads are compared for 10 and 500 µm ATP. Note that events were pooled using a bin width of 4 pN. Adequate event numbers (n) offered reliable estimates of the time constants and thereby detachment rates presented in panel E and F. For example, n_4−8_ indicates that events from force range 4 pN – 7.99 pN were pooled to generate the cumulative distributions and fitted with double exponential function to determine time constants (τ) and amplitudes (A) using MEMLET program. At 500 µm ATP, for resisting load n_0−4_ = 860, n_4−8_ = 1257, n_8−12_ = 1627, n_12−16_ = 279, for assisting load; n_4_ = 198, n_4−8_ = 265, n_8−12_ = 403, n_12−16_ = 223, Total events = 5112, N = 54. At 10 µm ATP, resisting load, n_4_ = 189, n_4−8_ = 248, n_8−12_ = 201, n_12−16_ = 138, Assisting load; n_4_ = 142, n_4−8_ = 172, n_8−12_ = 154, n_12−16_ = 77, Total events = 1312, N = 20. n = number of events, N = number of myosin molecules.

The ADP‐bound actomyosin state has been shown to be load‐dependent for several myosin isoforms. For SolM‐II, we analyzed the interaction lifetime at a saturating ATP concentration of 500 µm ATP to examine the properties of the ADP bound state specifically. At this ATP concentration, the event duration is expected to be limited by the rate of ADP release. The motors experienced an external load range of ± 15 pN depending on the interaction distance from the laser trap. As shown in **Figure** **3**A,B, at lower assisting and resisting load (±5 pN) the interaction duration were shorter. With increasing resisting load the actomyosin interaction lifetime increased (Figure 3C), i.e., the detachment rate decreased. Surprisingly, the event lifetimes at higher assisting load also increased. In our knowledge, this increase in actomyosin‐bound duration under assisting load was never reported for myosin before. When the events within 4‐pN binwidth were pooled to derive the average time constants by using MEMLET program,^[^ ^23^ ^]^ it became apparent that the frequency distribution cannot be fitted with a single exponential function. Two exponential functions were required to adequately fit the events with wide lifetime distribution, suggesting two distinct populations (Figure 3D). The analysis yielded the time constants and corresponding fraction of events for two distinct populations. The time constants (τ_1_ and τ_2_) were used to estimate the detachment rates (1/τ), i.e., fast (*k_f_)* and slow (*k_s_*) detachment rates, respectively (Figure S5, Supporting Information). The faster detachment rates *k_f_* were more than tenfold different, i.e., 267 s^−1^ versus 30 s^−1^ at low and higher loads, respectively (Figure 3E; Figure S5A, Supporting Information). *k_s_* ranged between 20 and 1 s^−1^ at low and high loads, respectively. The dissociation rates between the two populations ranged from ≈7 to 32‐fold different at various loads (Figure S5A, Supporting Information). Interestingly, both *k_f_* and *k_s_* responded to increasing resistive as well as assistive load by slowing the actomyosin unbinding rates (Figure S5A, Supporting Information), exhibiting “catch bond” behavior, i.e., the duration of actomyosin bound state increased with load.

The measurements performed at 10 µm ATP were aimed at examining a strong‐bound rigor state, affected by load. At this ATP concentration, the rigor state dominates the bound AM duration, and dissociation is induced by ATP binding. The analysis of the events as described above again yielded two populations, with a fast population having the detachment rate (*k_f_*) of 243 s^−1^ that is comparable to the one observed at 500 µm [ATP] (Figure 3E; Figure S5B, Supporting Information). The slow detachment rates (*k_s_*) showed load dependence at 10 µm ATP ranging between 3.9 and 0.4 s^−1^ (Figure 3F). Notably, the distribution of the fractions corresponding to the fast dissociation rates at 10 and 500 µm ATP were comparable. As shown in Figure 3G, the fraction corresponding to the fast detachment rates ranged between ≈70% and 90% and remained constant throughout different load conditions. Thus, the slow population represented ≈10–30% of the binding events at both low and high ATP concentrations. Collectively, actomyosin detachment rates were load dependent, and only the second population (A_s_) corresponding to *k_s_* was responsive to ATP concentration, while the fraction of events (A_f_) corresponding to a faster rate of detachment, *k_f_* remained ATP concentration independent.

Note that the fast population with average detachment rates of 267 or 243 s^−1^ could not be observed in our measurements at low load range with the stationary trap, as the time resolution was insufficient.

### 2.5 Actomyosin Detachment Kinetics for β‐Cardiac Myosin are Load‐Dependent

**Figure 4:**
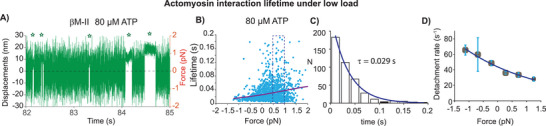
Actomyosin detachment kinetics of βM‐II. A) A representative record from stationary optical trap set up with bead displacement over time trace displaying intermittent βM‐II interaction with actin at 80 µm ATP. Discernible interaction events marked with stars observed on positive displacement side in the data trace. Force values are displayed on the right Y‐axis scale. B) Scatter plot showing individual actomyosin interaction lifetime events measured at load range of −2–+2 pN using stationary trap setup. Longer lifetime events have been observed with increasing resisting load. Data was fitted with the single exponential (fit shown as a purple line) as described in Figure 1D to estimate “*k*_0_” and “d”, N = 5, n = 1482. C) Histogram of lifetimes of the binding events. Events shown in dotted rectangle in Figure B are binned, n = 541. Maximum likelihood estimation (MLE) was employed to calculate the average lifetime (τ) and thereby detachment rate (1/τ) of 33.5 ± 2.78 s^−1^, n = 422. D) Detachment rates over force plot. Binwidth of 0.5 pN was applied to pool the events within corresponding force range. Events in 1–2 pN force range were pooled for the last data points. Plot fitted with user defined single exponential function (Arrhenius equation): *k* = *ko* exp (‐F*d/*k_B_* *T) in origin lab tool, yielded *k_0_* of 43.76 ± 1.07 s^−1^ and d of 1.56 ± 0.12 s^−1^.

Our earlier studies showed differences between the SolM‐II and βM‐II in actin filament gliding speeds. Corresponding to the threefold slower velocity, the SolM‐II displayed threefold reduced ADP release rate than βM‐II, i.e., 30 s^−1 ^versus 88 s^−1^, respectively.^[^ ^19^ ^]^ Here, we probed the dependence of actomyosin detachment kinetics for β‐cardiac myosin at low and higher load. First, we examined binding events at 80 µm ATP under low load range of ± 2 pN. **Figure** **4**A shows displacement over time record with intermittent interactions between actin filament and individual myosin molecule. Again, under this condition, the events appear biased to the positive force side, i.e., under resistive load. Due to the lower temporal resolution, the detection of actomyosin interaction events on negative or under assistive load are underestimated. Event durations prolonged with increasing resisting load, as shown in Figure 4B. Analysis for the load‐dependent detachment rate fitted with the approximation of Arrhenius equation for βM‐II yielded “*k_0_*” of 41 ± 2.6 s^−1^ and “d” of 1.87 ± 1.4 nm at 80 µm ATP. As described in earlier section (cf. Figure 1), we further estimated “*k_0_*” and “d” from the binned events for specific load range Figure 4C,D. The resulting parameters were comparable to those obtained from fitting the scatter data plot. Earlier, from the ATP concentration dependence of the detachment rate, we estimated the maximum detachment rate of ≈88 s^−1^.^[^ ^19^ ^]^ The current estimate of the detachment rate at zero load is nearly two times lower for βM‐II, indicating that measurements at higher than 80 µm ATP concentrations would be necessary to determine the maximum actomyosin dissociation rate that is limited by the rate of ADP release. Due to low time resolution with the stationary trap setup, measurements beyond 80 µm ATP were not possible. Nevertheless, a “d” value of ≈1.87 nm indicated that the actomyosin detachment rate is load‐dependent for βM‐II. Recently, we reported an overall stroke size of ≈5.33 nm, comprising the first powerstroke (δ1) of 4.49 nm and the second powerstroke (δ2) of 0.84 nm for βM‐II.^[^ ^19^ ^]^

**Figure 5:**
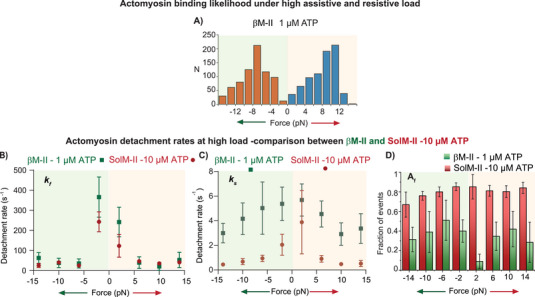
A) For βM‐II, actomyosin binding likelihood during actin filament excursion in alternating directions using square wave scanning at 1 µm ATP; n = 733 and 713 on the resisting and assisting sides, respectively, yielding a ratio of 1.02. B,C) Actomyosin detachment rates are compared between βM‐II at 1 µm ATP and SolM‐II at 10 µm ATP. For βM‐II, events were pooled for 4‐pN binwidth, and the average lifetime (τ) was determined by fitting the events with two exponential fits as explained in Figure 3D. Reciprocal of time constants, i.e., detachment rates (*k_f_* and *k_s_*) estimated and plotted over external force. The detachment rates were plotted at increasing resisting and assisting load. D) The fraction of events (A_f_) corresponding to first population (i.e., *k_f_)* at various loads are compared for βM‐II and SolM‐II. At 1 µm ATP; resisting load n_4_ = 101, n_4−8_ = 206, n_8−12_ = 344, n_12−16_ = 101, Assisting load; n_4_ = 91, n_4−8_ = 205, n_8−12_ = 329, n_12−16_ = 109, total events = 1486, N = 30 n = number of events, N = number of myosin molecules.

We further extended our investigations to examine the load sensitivity at higher load using the square wave‐scanning mode at 1 µm ATP. At this concentration, we expected to observe the actomyosin binding events that primarily represent the lifetime of the rigor state; the cues were taken from our recent report.^[^ ^19^ ^]^ The binding likelihood at 1 µm ATP was observed to be equal on both sides (**Figure** **5**A). After establishing the actin filament polarity as described in earlier section for SolM‐II, the square wave routine was applied to measure the actomyosin bound durations at higher loads of ± 15 pN. Similar to our observation for SolM‐II, the event lifetimes were adequately fitted with two exponential functions yielding fast and slow detachment rates. At 1 µm ATP, the fastest detachment rate (*k_f_*) was ≈366 s^−1^ at low assisting load and load dependence was observed (Figure 5B). Both increasing resistive and assistive load slowed down the detachment rate (Figure 5B,C). *k_f_* ranged between 366 and 20 s^−1^, whereas *K_s_* between 5.7 and 2.9 s^−1^. While *k_f_* displayed a strong load sensitivity, the change in *k_s_* remained modest by about twofold. The fractions of the fast component were plotted to compare the populations contributing to *k_f_* (Figure 5D). The fraction remained nearly constant between 30% and 50% except for the resisting load of 2‐pN where only 10% contributed to the faster population.

The measurements for βM‐II at saturating ATP concentration could not be performed. Higher ATP concentration (>100 µm) and a faster detection system would be required to gain knowledge of relevant intermediate states for βM‐II.

### 2.6 Comparison Between βM‐II and SolM‐II

At low ATP concentration, two distinct populations *k_f_* and *k_s_* were observed for both SolM‐II and βM‐II. Both myosins showed similar load response trend for *k_f_* (Figure 5B,C). βM‐II detached ≈100 s^−1^ faster under the low assisting load condition (*k_f_* of 243 s^−1^ for SolM‐II vs 366 s^−1^ for βM‐II). Interestingly, the fractions of events that contributed to the two populations of events were markedly different, with βM‐II showing lower population, i.e., 10–50% against 70–90% for SolM‐II that is responsible for the *k_f_* (Figure 5D)*_._* This may be an indication that the actomyosin transition state responsible for fast detachment rates transitioned to the next state in the cross‐bridge cycle with a higher probability for βM‐II as compared to SolM‐II.

## 3 Discussion

**Figure 6:**
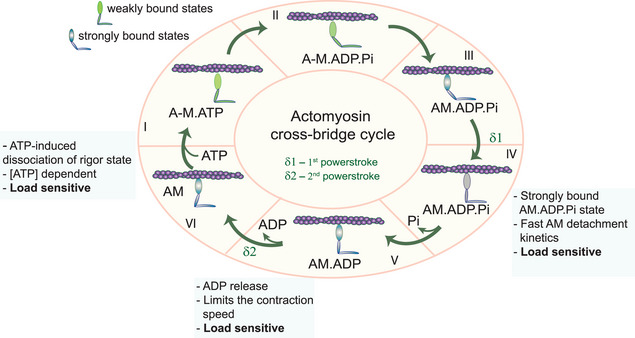
Actomyosin crossbridge cycle. Key intermediate states are shown. As the myosin interacts with actin, strongly bound transition states are indicated (blue myosin heads). Our proposed post‐powerstroke AM.ADP.Pi state is indicated with a grey motor head. The three load‐responsive states identified in our studies as are shown. Only single myosin heads are shown. A – Actin, M – myosin. A‐M indicates weak interaction between actin and myosin, AM – strong interaction.

In summary, our studies reveal previously unidentified actomyosin conformations sensitive to load. The main findings in this study were: i) The higher assisting load ≥5 pN increased the actomyosin‐attached lifetimes, which was mainly observed under resisting load in previous studies. ii) Besides the ADP‐bound actomyosin state, we identified additional force‐generating intermediate states, responding to a range of loads. iii) At least three load‐sensitive cross‐bridge states that conceivably correspond to different conformations of the actomyosin complex, with the varied extent of a strong association between actin and myosin, were identified. We interpret these three states as: 1) actomyosin ADP.Pi strongly bound, 2) ADP‐bound, and 3) ATP‐ induced actomyosin dissociation or rigor state (summarized in **Figure** **6**). The new features, i.e., load sensitive actomyosin ADP.Pi state and reduced actomyosin dissociation at high assisting and resisting loads were inferred for both SolM‐II and βM‐II. With our new observation, the earlier model that the rate of ADP release determines the shortening velocity^[^ ^10^, ^24^, ^25^, ^26^ ^]^ can be redrafted to the duration of the force‐producing ADP.Pi and ADP state and the transition rate defines the shortening speed under load. The slowing down of the reaction rate coupled to Pi release was predicted earlier,^[^ ^27^ ^]^ but not experimentally shown in our knowledge. This study provides the first experimental evidence for this. In future studies, it would be interesting to see whether in other motor forms a load‐sensitive ADP.Pi bound states exists, or if it is rather myosin isoform‐specific.

### 3.1 Identification of Strong Binding AM.ADP.Pi State as Load‐Dependent State

We interpreted the state independent of ATP concentration but dependent of load to be the ADP.Pi strongly‐bound actomyosin transition state that was observed both for SolM‐II and βM‐II, albeit with slight variations in detachment rates. The force‐producing AM.ADP.Pi state in the current report is likely to be a stereo‐specific, post‐first‐powerstroke state. The newly identified load‐sensitive state is distinct from the previously observed short‐lived and assumed pre‐powerstroke, actin cleft open state.^[^ ^28^ ^]^

For SolM II, within a load range of zero to ± 15 pN, the detachment rate, *k_f_*, corresponding to this state reduced from 243 to 25 s^−1^, while for βM‐II, from 366 to 35 s^−1^. The reason for the interpretation of this state being a strongly bound AM.ADP.Pi state is that in our experimental set up the state must be strongly bound to be detected as an event. The weak binding states exhibited an order of magnitude faster rate of actomyosin detachment in the range of 10^3^ s^−1^, as earlier reported.^[^ ^28^ ^]^ In contrast to our observations of reduced detachment rate at higher loads, increased rate of detachment was noted for the short‐lived weak binding states and intermediate states, which were described by slip bond model. Our time resolution is below the detection range of the weak binding states. Besides, the Pi release rate is estimated to be ≈400 s^−1^ for recombinant human beta cardiac HMM,^[^ ^28^ ^]^ which closely matches with our *k_f_* of 366 s^−1^ under low load for native βM‐II. As the myosin proceeds through the cross‐bridge cycle, there are two possible outcomes, i.e., i) to attain the next transition state (e.g., from strongly bound ADP.Pi to ADP state) or ii) to detach in the ADP.Pi state. Accordingly, it can be inferred that *k_f_* corresponds to the Pi release rate. Whether the presumed strongly bound ADP.Pi bound actomyosin state is a pre‐ or post‐powerstroke state needs further investigation. One of the unresolved ambiguities in the field is the sequence of powerstroke and Pi release during the cross‐bridge cycle. While some studies support the Pi release precedes powerstroke,^[^ ^28^, ^29^, ^30^ ^]^ other studies interpret that powerstroke can take place before Pi release.^[^ ^9^, ^31^, ^32^ ^]^ In a context of load dependence, 1) if Pi is released after the powerstroke, the occurrence of the powerstroke will not be delayed under load, i.e., the stroke occurs after immediate binding, 2) if Pi releases before powerstroke, the powerstroke will be delayed, 3) if Pi release and powerstroke occur simultaneously, both are expected to be delayed. From direct measurement of initial events in force generation by myosins, the stroke was reported to occur within 200 µs after binding, even at loads up to 4.5 pN.^[^ ^28^ ^]^ Furthermore, the transition rate of pre‐powerstroke, stereospecificailly bound AM.ADP.Pi state to post‐powerstroke strongly bound state was estimated to be equivalent to the stroke rate in the range of ≈1000 s^−1^. If this is the case, combined with our observation with dissociation rate (including Pi release) of 366 s^−1^, and a stroke rate of 1000 s^−1^,^[^ ^28^ ^]^ it can be inferred that the Pi is released after the powerstroke. Woody et al. further reported that the stroke rate was increased from 700 to 5250 s^−1^, with increasing force in the range of ±5 pN. However, the amplitude of stroke size reduced with increasing force.^[^ ^28^, ^33^ ^]^ This effect on the powerstroke size, i.e., reduction under resisting versus assisting load was also observed in muscle fibers.^[^ ^34^ ^]^ Since our measurements cannot resolve the initial actomyosin binding and stroke size in the cross‐bridge cycle, the effect on the stroke size could not be verified. Studies combining first powerstroke measurement or initiation of powerstroke relative to Pi release rate that matches with observed actomyosin dissociation rate (366 s^−1^) in this study would be essential to further verify this proposal of sequence of events in the ATPase cycle.

In earlier studies by Kraft et al.,^[^ ^35^ ^]^ using AlF4 as an inorganic Pi analog, two conformations of ADP.Pi states were captured. The observed states ADP.AlF4‐I and ADP‐AlF4‐II were interpreted as states corresponding to the weakly‐ and strongly‐associated actomyosin complex. ADP.AlF4‐I showed properties characteristic of the weak binding conformation of the myosin head that is consistent with the pre‐power‐stroke structure of the catalytic domain.^[^ ^36^ ^]^ A conformation in ADP.AlF4‐II state showed a tighter and stereospecific binding between the myosin head and the activated thin filament. These results implied that while phosphate is still present in the active site, the structural elements within the motor domain rearrange from weak, non‐stereospecifically attached conformation to that of tight stereospecific connection to the thin filament. Furthermore, it was proposed that the transition to the strong binding state is essential before the Pi release and powerstroke.^[^ ^37^ ^]^ Our proposed strongly‐bound actomyosin ADP.Pi state is consistent with the ADP‐AlF4‐II bound conformation observed in Kraft et al.^[^ ^35^ ^]^

The rate of ADP release estimated by the maximal actomyosin dissociation limited by ADP release is 30 s^−1^ for SolM‐II and 88 s^−1^ for βM‐II.^[^ ^19^ ^]^ These rates are consistent with solution kinetics and single molecule studies for SolM‐II and cardiac myosin.^[^ ^14^, ^38^ ^]^ Actomyosin·ADP complexes with two proposed AM.ADP states for slow myosin detached in the range of 20–6 s^−1^ respectively.^[^ ^38^ ^]^ Therefore, the identified intermediate state that dissociates in the range of 267–366 s^−1^ for SolM‐II and βM‐II, respectively, is unlikely to be one of the ADP‐bound states. We assume that the ADP.Pi‐ bound myosin may possess lower affinity for actin than the ADP‐bound myosin state since the rate that corresponds to the ADP release is nearly tenfold slower at low load (cf Figure 5B,C). Note that for SolM‐II, at 500 µm ATP, *k_s_* representing the ADP‐bound actomyosin complex detached with a rate of ≈20 s^−1^ at low load. For this fraction of events detachment rate reduced from 20 to 2 s^−1^, i.e., by about tenfold at high load. Thus, the ADP bound actomyosin complex was found load‐dependent for SolM‐II as indicated in the measurements at 500 µm [ATP].

Altogether, from our measurements and circumstantial evidence from earlier studies it can be inferred that the observed state may be AM.ADP.Pi strongly‐bound post‐powerstroke state. The state is indicated in the Figure 6 (as state IV) in the cross‐bridge cycle. The detachment kinetics of this state are slower than the assumed weak binding ADP.Pi state and about ten times faster than the strong actomyosin ADP‐bound state.

It's apparent that Pi release rates estimated from the actomyosin detachment rates in previous^[^ ^28^ ^]^ and our current single molecule studies, i.e., 400 and 366 s^−1^, respectively, are much faster than the estimated Pi release rate of 11.6 − 17 s^−1^ for cardiac myosins in solution studies.^[^ ^39^, ^40^ ^]^ The discrepancy is likely to be a result of how Pi release rate is defined in different studies. For example, our estimates primarily indicate the Pi release from strongly bound actomyosin ADP.Pi intermediate state. In solution studies, multiple pre‐Pi release states, i.e., the rate of ATP binding, hydrolysis, and then Pi dissociation contributes to the observed Pi release rate. Second, the existence of low‐load conditions in solution experiments more likely favor the dissociation of actomyosin both in short‐lived A‐M.ADP.Pi state (presumably pre‐first powerstroke) reported in Woody et al.^[^ ^28^ ^]^ and force‐producing AM.ADP.Pi states (presumably post‐first powerstroke) observed in the current study. Thus, there is a higher probability of actomyosin dissociation without Pi release and completing the cross‐bridge cycle. Note that in both the studies, for  βM‐II the population corresponding to fast dissociation rate was in the range of 10–50%. For SolM‐II, this population was even higher at 80–90% under low load (cf. Figure 5D). These factors seemingly contribute to lower overall Pi release rate estimates in solution kinetics experiment.

Another important aspect recognized from this finding is how load guides the cross‐bridge from AM.ADP.Pi to AM.ADP state, which may vary in distinct myosin forms. It appears that under low or no load conditions, in ADP.Pi conformation, actomyosin can undergo faster dissociation (≥366 s^−1^ for βM‐II). Increase in load prolongs the strong‐bound force‐generating ADP.Pi state. In other words, the load may contribute to capturing the AM.ADP.Pi state in the crossbridge cycle, presumably due to increased energy barrier for reverse as well as forward reaction (catch‐bond configuration). This reasoning is based on the observation that the fraction of events contributing to the fast states (A_f_) remained nearly constant through the range of loads. We expected that faster Pi release would appear as decreasing A_f_ with increasing load. The higher dissociation rate in AM.ADP.Pi state (366 s^−1^) compare to AM.ADP state (≈88 s^−1^) for βM‐II,^[^ ^19^ ^]^ indicates that myosin has higher affinity for actin in ADP‐bound state than in ADP.Pi‐ bound configuration. Altogether, it appears that there is a higher probability of actomyosin dissociation in the Pi‐bound state than in the ADP‐bound state. Conceivably, under in vivo condition, the load dependent dissociation further helps in fine‐tuning sustainable energy usage by moderating the attached myosin heads as per physiological demand.

### 3.2 Resistive Versus Assistive Forces

The kinetics of mechanical transition states depend on the load. Previous studies on various myosins showed that the effect of load on the lifetime of the bound crossbridge is to delay the actomyosin dissociation under high resistive load, but to accelerate under low assisting load. In this study, the cross‐bridges showed rather different properties. Under low assisting load, the dissociation was accelerated, but under high assisting load, prolonged actomyosin‐bound states were observed. Although the total number of such events were lower than the ones observed under resisting load at higher [ATP] (3.7x for SolM‐II), the observation that under high assisting load the dissociation is delayed is noteworthy. The load affecting the conformation of myosin head domain at the actin binding interface under assisting load must be distinct from when pulled in the opposite direction as experienced in resisting load.

### 3.3 Rigor State and Load Sensitivity

Contrary to the previous report on the fast skeletal muscle myosin that suggested an accelerated detachment rate under resistive forces in the rigor state,^[^ ^33^ ^]^ we observed a decrease in the detachment rate with increasing resistive as well as assistive force for SolM‐II (by about tenfold) and rather modest effect on βM‐II (by about twofold). These results may suggest isoform‐specific differences in load response among various myosin forms. Under high external load, it is likely that in the rigor state, ATP binding is hindered/not favored due to the inaccessible ATP binding site delaying ATP‐induced actomyosin dissociation. In vivo implication of this state is rather unclear if the high load can distort the myosin head in a way that ATP cannot associate, thus further delaying the actomyosin dissociation. Less than 20% of the events in this state at low ATP concentration in our measurements indicate that the fraction corresponding to this state is rather low. Furthermore, at high ATP concentration under physiological condition the existence of this population may not be as substantial.

### 3.4 Weak Binding State and Load Sensitivity

Weak‐binding conformation of myosin head is characterized by the presence of ATP or hydrolysis product ADP.Pi in the active site and the cleft at the interface of the actin‐binding site in the open state.^[^ ^32^, ^41^ ^]^ Previously, using an ultrafast force‐clamp optical trap, different populations of states with a lifetime of <1 ms assumed to be weak binding states were registered.^[^ ^33^ ^]^ The initial binding between the actin and myosin, i.e., weak attachment and detachment at moderate to high load in these transition states, is beyond our detection limit. Our current setup allows the identification of event populations with strongly bound actomyosin states, mainly. The strong binding conformation are assumed stereospecific interactions between myosin head and actin.

### 3.5 βM‐II and SolM‐II

Transition from presumed actomyosin ADP.Pi to ADP state appears to occur with a higher probability for βM‐II as noticeable from the population of events. We observed different fractions of binding events for fast population, i.e., 80–90% for SolM‐II versus 10–50% for βM‐II in presumed ADP.Pi bound states at various loads (cf Figure 5D). The fast detachment rates *k_f_* are different by ≈100 s^−1^ between the two myosins at low load. This also implies the individual differences in the chemomechanical properties, i.e., the duration in each transition state and their varied sensitivity to load.

We cannot rule out intermixing within different populations in measuring the fraction of events and the duration of individual transition states, i.e., a small fraction of presumed ADP.Pi state may contain ADP states and vice versa. For SolM‐II the dissociation rates at high loads around ± 14 pN at 10 and 500 µm ATP are almost identical and thus challenging to classify these as actomyosin.ADP or rigor state events. For SolM‐II, our current temporal resolution enabled the use of low and high ATP concentrations, allowing us to distinguish apparent actomyosin ADP.Pi and ADP states with higher precision. However, load‐dependence measurements for β‐cardiac myosins at saturating ATP concentrations were not possible. Future investigations under this condition will be key to identifying the distribution of actomyosin ADP.Pi and ADP‐populated states to elucidate the in vivo implications of the load‐dependent mechanisms. The regulatory components on actin filaments, i.e., the troponin‐tropomyosin complex play a critical role in cross‐ bridge cycling and are highly relevant for load‐dependent changes on actomyosin interactions in vivo. It remains to be seen whether the mechanosensitive states observed at single molecule level are analogous in the higher order system including regulated thin filaments.

### 3.6 Advantages and Shortcomings of the Current Study Approach

We employed full‐length myosin in this study instead of single‐headed myosin (subfragment‐1), which is typically used in such experiments. Proteolytic digestion of the native myosin is associated with loss of light chains (either MLC1 or MLC2) depending on the digestive enzymes (papain or chymotrypsin) chosen for generating S1 motor domains. We previously showed that the light chains participate in the fine‐tuning of myosin function^[^ ^19^, ^42^, ^43^ ^]^ and, therefore, assume that truncation or complete loss of either of the light chains may affect the motor function. Validation of the integrity of the motor complex with the correct and full length LCs would be critical to compare the truncated versions with the native motor composition. On the other hand, a dimeric full‐length motor has a probability that both heads of a myosin molecule may interact with the actin filament. However, single‐molecule level studies from our group^[^ ^21^ ^]^ and previous indications from muscle fiber studies support a single myosin head interaction with actin during the cross‐bridge cycle.^[^ ^44^, ^45^ ^]^ Consistent with our earlier studies, in the analyzed data traces of individual binding events in this study, we did not observe any indications of simultaneous interaction of both myosin heads with the actin filament. Such a possibility, though, cannot be excluded entirely. Nevertheless, in a dimeric motor, the unbound head may affect the function of the myosin head that is directly interacting with the actin filament; this arrangement is comparable to physiological conditions.

With our optical trapping combined with the square‐wave scanning approach, the effect of higher external forces of up to 15 pN on the actomyosin cross‐bridges can be probed. The 3‐bead configuration is well suited for the investigation of the non‐processive motors. Another advantage over the previously established method, i.e., harmonic force spectroscopy (HFS)^[^ ^46^ ^]^ is that our experimental setup allows the initial interaction between actin and myosin to be probed to determine the binding probability of myosin to actin under assisting versus resisting load. Besides, a constant force is applied to the myosin heads during strong interaction with actin. However, our set up is not as fast as the currently available ultrafast force spectroscopy (UFFC) setup,^[^ ^33^ ^]^ which additionally benefits from a positive feedback option to control the applied load. Our measurements require a considerably stable actin filament that can sustain several cycles of rapid excursions for sufficient lengths of time to collect binding events under different load ranges for the same myosin molecule. The actin filament stability remains one of the major limiting factors.

### 3.7 Implication of Load Dependence Studies

In‐depth knowledge of the force‐sensitive response of individual states in diverse myosins helps us understand the fundamental mechanisms whereby myosins accomplish respective intracellular tasks. For example, in sarcomeres, load‐dependent change in detachment kinetics and mechanics provide a means to respond to the changing physiological requirements of the distinct muscle. Load‐dependent response of myosin molecules simultaneously engaged with an actin filament (e.g., in a sarcomere) may further promote coordinating the action of molecules over long stretches without a need for direct intermolecular interaction. An allosteric communication is thus possible among individual motors. Precise mechanistic details enhance our understanding of the dysfunctional motor forms. For example, cardiomyopathy‐causing mutations in ventricular myosin heavy chain and the small molecule compounds employed to correct the functional defects can modulate the load‐dependent detachment kinetics of single myosin molecules as reported earlier.^[^ ^15^ ^]^ The site of mutations within a myosin molecule may affect different intermediate states in the cross‐bridge cycle, and thereby load dependent kinetics. Therefore, crucial insights into relevant mechanosensitive kinetics and mechanical states are expected to facilitate the development of specifically targeted and thus effective treatments for numerous myosin‐associated diseases.

## 4 Experimental Section

### Tissue

The muscle and cardiac tissues were collected from New Zealand white rabbits, Crl:KBL (NZW). The animals were euthanized as per the guidelines from German animal protection act §7 (sacrifice for scientific purposes). In this study, shared organs originating from the animals approved for experiments with authorization number 18A255 were used. The animals registered under reference number G43290, were obtained from Charles River France. All the procedures were carried out in accordance with relevant guidelines and regulations from the Lower Saxony State Office for Consumer Protection and Food Safety and Hannover Medical School, Germany.

### Native Myosin II

Rabbit ventricular tissue and *M. soleus* were excised, cut into smaller pieces and stored in liquid nitrogen. Full length cardiac myosin II (βM‐II) and slow skeletal myosin (SolM‐II) were extracted by homogenizing the frozen tissue and incubating the tissue powder in the high salt extraction buffer (0.5 m NaCl, 50 mm HEPES, pH, 7.0, 5 mm MgCl_2_, 2.5 mm MgATP, and 2 mm DTT) as previously described.^[^ ^47^, ^48^ ^]^ The cardiac myosin was isolated from 50 to 100 mg ventricular tissue powder for each experiment as described in^[^ ^49^ ^]^ with some modifications. Isolated myosin was aliquoted, flash frozen in liquid nitrogen and stored at −80 °C in 50% glycerol.

### Preparation of Actin Filaments

To obtain sufficiently longer biotinylated actin filaments (≥20 µm) for optical trapping experiments, chicken G actin and biotinylated G actin was mixed in equimolar ratios to a final concentration of 0.1 µg µl^−1^ each in p‐buffer (5 mm Na‐phosphate, 50 mm K‐acetate, and 2 mm Mg‐acetate), containing 1 mm DTT, 1 mm ATP, and 0.5 mm AEBSF protease inhibitor (Cat No. 30827‐99‐7, PanReac Applichem ITW). The mixture was incubated overnight at 4 °C, and followed by addition of equimolar concentration of fluorescent (TMR, tertramethylrhodamine) phalloidin (cat no. P1951, Sigma–Aldrich) and biotin phalloidin (0.23 nm, Invitrogen/Thermofischer Scientific, B7474) to label the actin filaments.

### 3‐Bead Assay – Stationary Optical Trap Mode

The optical trapping set up was described in detail previously.^[^ ^50^, ^51^ ^]^ For the assay, flow cells with ≈15 µL chamber volumes were assembled using coverslips with nitrocellulose‐coated beads. Glass microspheres (1–1.5 µm) suspended in 0.05% nitrocellulose in amyl acetate were applied to 18×18 mm coverslips. All the dilutions of biotin‐actin filaments were made in reaction buffer (KS buffer) containing 25 mm KCl, 25 mm Hepes (pH 7.4), 4 mm MgCl_2_, and 1 mm DTT. The full‐length native myosin was diluted in high salt extraction buffer without MgATP. For the experiment, the chamber was prepared as follows, 1) flow cells were first incubated with 1 µg mL^−1^ native myosin for 1 min, 2) washed with high salt extraction buffer without ATP and thereafter with KS buffer, 3) followed by wash with 1 mg mL^−1^ BSA and incubated further for 2 min to block the surface, 4) finally, reaction mixture containing 0.8 µm neutravidin coated polystyrene beads (Polyscience, USA) and 1–2 nm biotinylated actin was flowed in with 10 µm ATP (or varied concentrations of ATP), ATP regenerating system (10 mm creatine phosphate, and 0.01 unit creating kinase) and deoxygenating system (0.2 mg mL^−1^ catalase, 0.8 mg mL^−1^ glucose oxidase, 2 mg mL^−1^ glucose, and 20 mm DTT). The assembled flow chamber was sealed with silicon and placed on an inverted microscope for imaging and trapping assay.

An actin filament was suspended in between the two laser trapped beads (Figure 1A), pre‐stretched, and brought in contact with the platform bead immobilized on the chamber surface. Low‐compliance links between the trapped beads and the filament were adjusted to ≈0.2 pN nm^−1^ or higher.^[^ ^52^ ^]^ The bead positions were precisely detected with two 4‐quadrant photodetectors (QD), recorded and analyzed. The acto‐myosin interaction events were monitored as a reduction in free Brownian noise of the two trapped beads. Data traces were collected at a sampling rate of 10 000 Hz and low‐pass filtered at 5000 Hz. All the experiments were carried out at room temperature of ≈22 °C.

### 3‐Bead Assay – Square Wave Scanning Mode

The two laser beams were synchronized to move at constant speed in a square waveform (Figure 2A). The laser beams were passed through EODs and set to move in a square waveform controlled via a waveform generator (National Instruments). The actin filament holding beads forming a dumbbell followed trap movements. The rapid movement of beams were followed by defined pause durations. The actin filament was thus moved in alternating directions across the platform bead holding a myosin molecule. As the bead followed the laser trap, the dumbbell velocity was 180 nm/0.5 ms. The frequency of the square wave was 0.1 to 1 Hz. Different frequencies were used to accommodate the actomyosin interaction events with durations of varied lifetimes, i.e., long ones at low ATP to shorter events at higher ATP concentrations. Force/load experienced by the motor was determined from the site of binding event during the excursion of the bead. The displacement from the binding site to the next forward bead position multiplied by the trap stiffness yields the force exerted on the motor. Trap stiffness was measured using equipartition method as described earlier.^[^ ^53^ ^]^ Positive feedback was not used in the square wave scanning method. For the force calculation, the combined trap stiffness was not used, but the stiffness of one side of the trap. As shown in Figure S3 (Supporting Information) the myosin interaction with actin disconnect the two sides of the dumbbell. Therefore, the myosin experiences the load from one side of the trap, either in the direction of pull (assisting load) or the opposite direction of myosin power stroke (resisting load). During the forward movement of the laser/bead, the trailing bead buckles while the leading bead experience the force from the laser. Thus for the assisting load, the trap stiffness from bead‐2/channel 2 was considered for force calculation while for the resisting load, trap stiffness from bead‐1/channel 1 is taken into account as shown in Figure S3 (Supporting Information).

### Data Analysis

Data records acquired with stationary trap mode were analyzed using the running variance and threshold method as described in Molloy et al.^[^ ^54^ ^]^ Actomyosin interaction events detected as reduction in noise were identified and Matlab routine was employed to analyze their bound duration “*t_on_*”. This method allowed the actomyosin bound states (low variance) to be distinguished from the unbound states (high variance). A threshold value was chosen from variance histogram to identify the start and end of an interaction event. Such analysis approach was comparable to variance‐Hidden‐Markov‐method^[^ ^55^ ^]^ of event detection from noisy data records. The data traces acquired using square wave scanning mode were analyzed manually for the lifetime of the actomyosin interaction. The beginning and end of these binding events show a clear distance from the stationary states of actin dumbbell (cf. Figure 2C; Figure S4, Supporting Information). The detected binding events provide two key pieces of information: the distance of binding event to the actin filament's next stationary state (laser motion paused for predefined time) that was used to derive the force experienced by the motor and the duration of actomyosin interaction.

### Single Myosin Molecule Interaction with Actin Filaments in Optical Trapping Measurements

For single‐molecule optical trapping experiments, routine tests were employed to test the probability that each data record was derived from an intermittent interaction between a single myosin molecule and actin filament. The criteria used to maximize the possibility of single myosin molecule analysis included, 1) myosin density on the bead surface was adjusted by diluting the myosin solution, 2) among 8–10 beads scanned for the presence of motor on the bead, typically, one bead should interact with the dumbbell, 3) the data traces with distinct well‐resolved actomyosin binding events were included in the analysis, 4) closely spaced binding events or stepwise binding indicate multiple molecules simultaneously or consecutively interacting with the dumbbell and such data records were excluded from the analysis. From the Poisson distribution knowing the percentage of beads without motor, it estimate the likelihood of presence of more than 1 motor per bead to be ≈5%. Since the data traces with closely spaced events were excluded, the effective likelihood was less than 5%. In total 174 molecules including β‐cardiac and M. soleus myosin were analyzed.

### Statistical Analysis

The detachment rate values were expressed as mean ± SE (standard error of fit) when the frequency distribution was fitted with double exponential functions in MEMLET program. Poisson distribution was used to estimate the likelihood of single molecules interacting with actin filaments in optical trap measurements. The distributions were assumed to be different only when p < 0.05.

## Conflict of Interest

The authors declare no conflict of interest.

## Author Contributions

M.A. conceived the project and designed the experiments with inputs from T.W., and A.N.. T.W. performed optical trapping measurements and analyzed the data. T.K. was involved in the discussions. M.A. wrote the manuscript with assistance from T.W. and A.N. All the authors contributed to the editing of the manuscript.

## Supporting information



Supporting Information

## Data Availability

The data that support the findings of this study are available from the corresponding author upon reasonable request.
