# Supplementary material for: Single‐Molecule Investigation of Load‐Dependent Actomyosin Dissociation Kinetics for Cardiac and Slow Skeletal Myosin
Source: Small. 2024 Oct 7;20(51):2406865. doi: 10.1002/smll.202406865 (PMC11657034; doi:10.1002/smll.202406865)
Supplement: Supplementary file 1 — Supporting Information [file SMLL-20-2406865-s001.pdf]

NANO · MICRO
small

Supporting Information

for *Small*, DOI 10.1002/smll.202406865

Single-Molecule Investigation of Load-Dependent Actomyosin Dissociation Kinetics for Cardiac and Slow Skeletal Myosin

*Tianbang Wang, Arnab Nayak, Theresia Kraft and Mamta Amrute-Nayak**

Supporting Information

Single-molecule Investigation of Load-dependent Actomyosin Dissociation Kinetics for Cardiac and Slow Skeletal myosin

Tianbang Wang[1], Arnab Nayak[1], Theresia Kraft[1], Mamta Amrute-Nayak[1]*

[1] Institute of Molecular and Cell Physiology, Hannover Medical School, 30625 Hannover, Germany

Corresponding Author*: Mamta Amrute-Nayak
E-Mail: amrute.mamta@mh-hannover.de

This file contains Supplementary Figures S1-S5 and Figure legends

Data trace acquired with Stationary trap

Binding event detection using running variance and threshold method

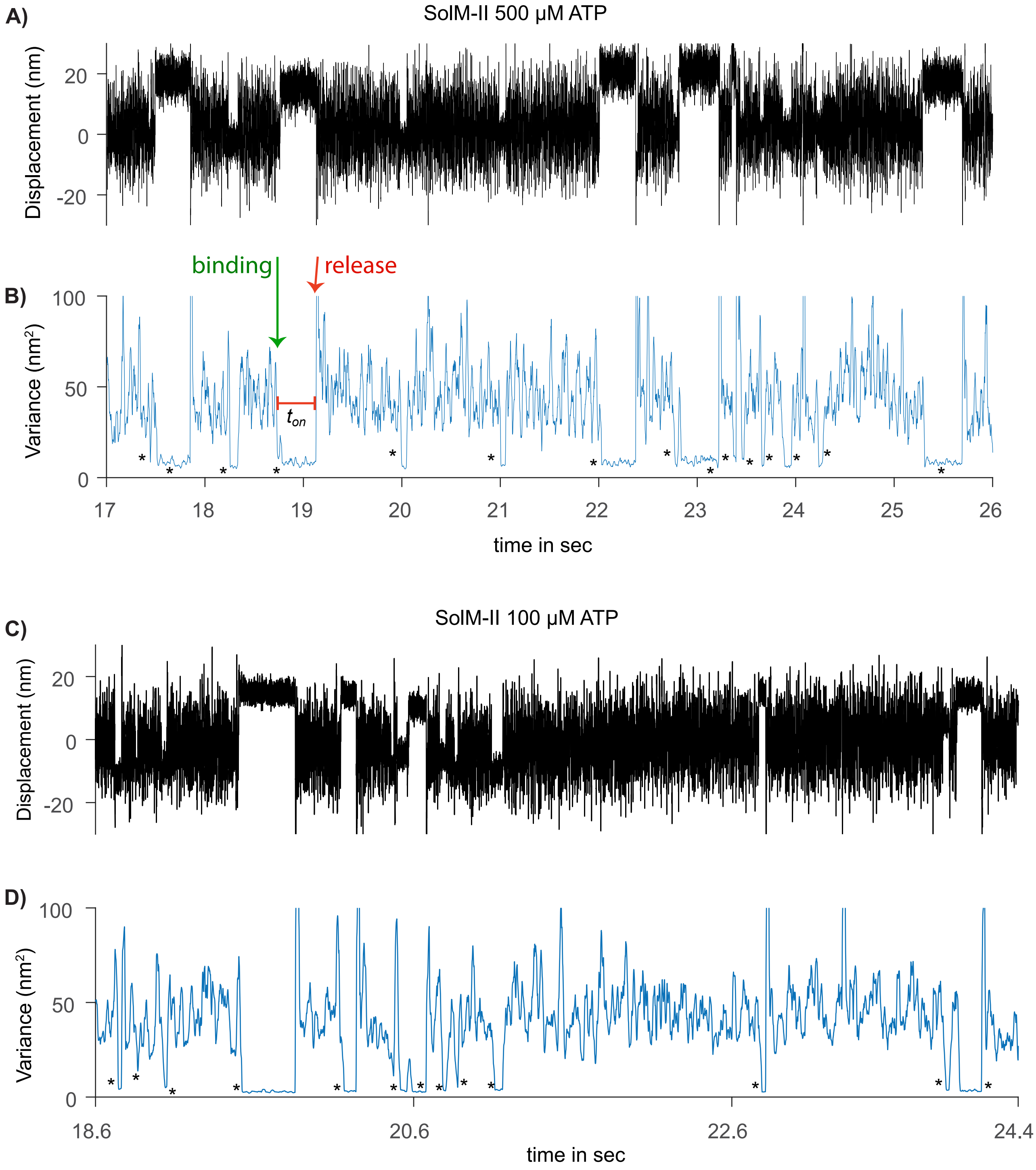

Figure S1. A and C) Original data trace acquired with stationary trap set up at 500 µM and 100 µM ATP. **B and D)** The variance of the displacement signal shown in A and C, respectively. The variance trace was generated by using a running window size of 50 data points. Subsequently, to enhance the signal-to-noise ratio of binding events, the variance was smoothened using a mean filter with a window size of 200 data points. The binding events are indicated with asterix in variance traces. AM binding and release are marked to show the duration of the bound state (t_{on})

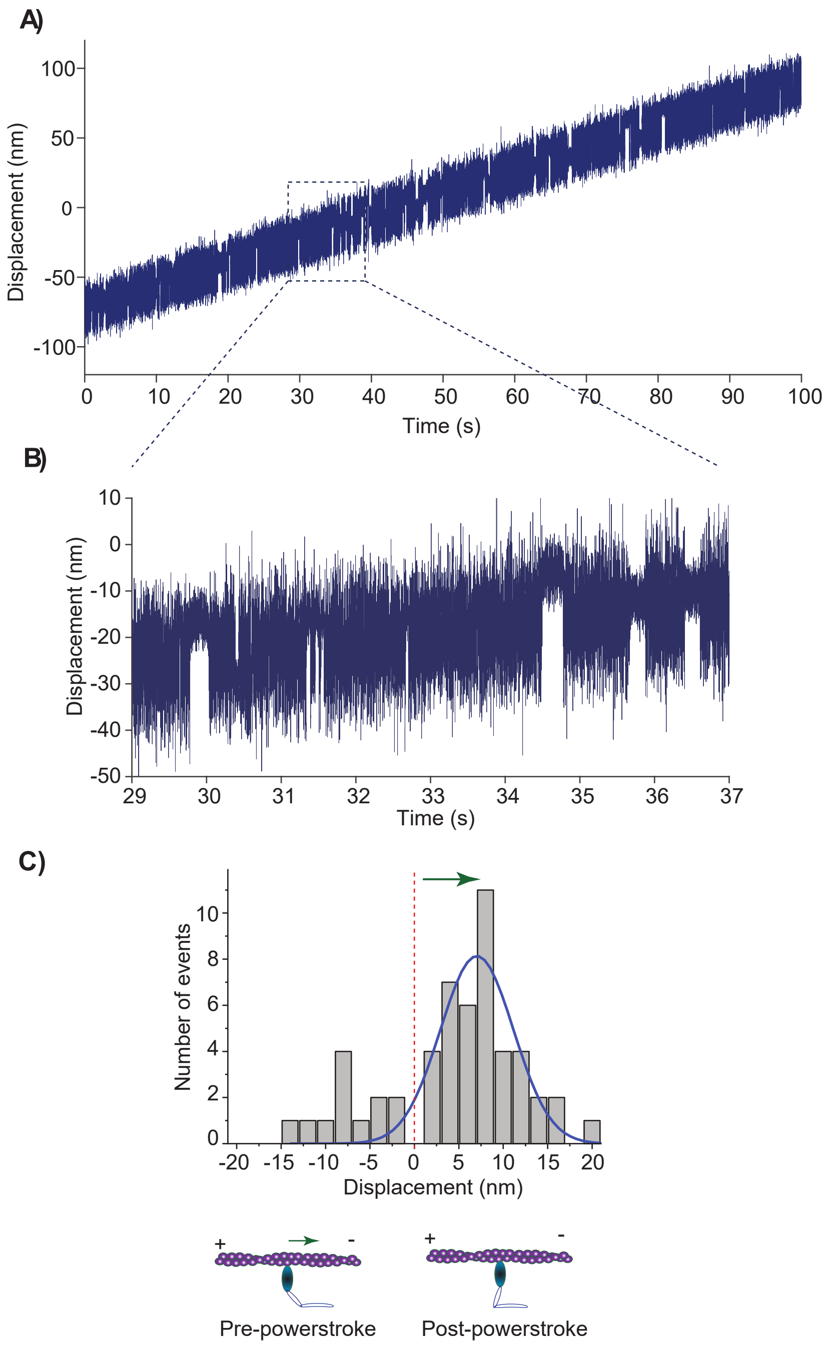

Figure S2. Determination of actin polarity. **A)** In a stationary trap set up, the dumbbell is moved across the pedestal bead such that the myosin molecule interacts with several actin monomers in a filament as shown in an original data trace of 100 s. **B)** The data trace from A is expanded to show several binding events. **C)** The histogram displays the displacement during the interaction events from the free dumbbell's mean position (0). Displacement for each individual event is measured from the mean free dumbbell position. The histogram shift method is used to establish the polarity of actin filaments. This method was used by Steffen et al to estimate the direction of the powerstroke from where the plus and minus end of the actin filament can be inferred [47]. As shown in a schematic below, the myosin associates at plus end and displaces the actin towards minus end. The direction of the shift thus indicates the minus end of actin filament.

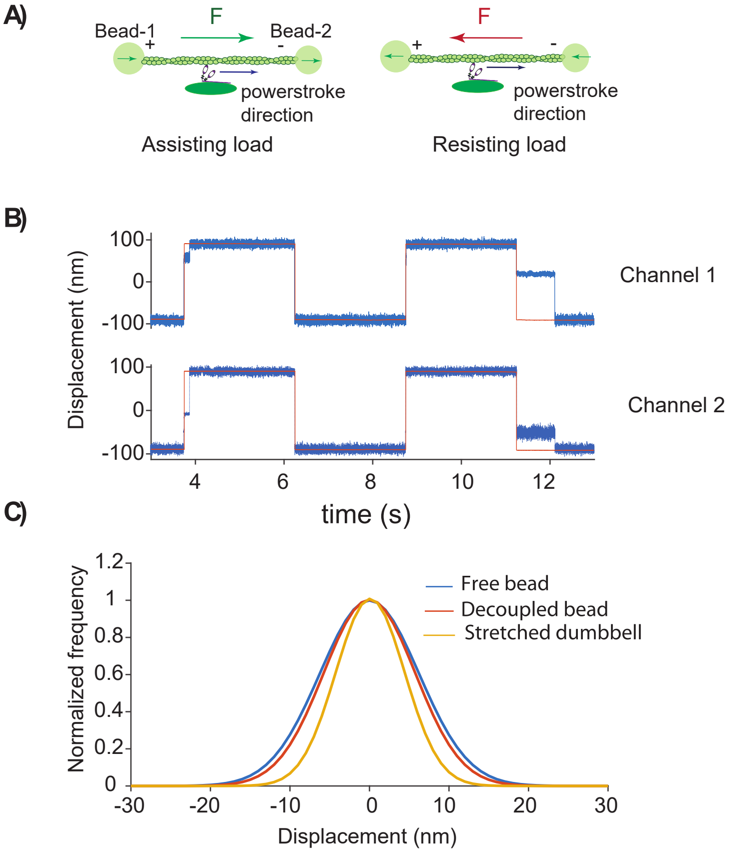

Figure S3. A) Illustration of the positive or negative load experienced my myosin. **B)** Original data record from a dumbbell with both channels, i.e., shadows of both beads 1 and 2 (blue trace) were collected after applying square wave mode. Actomyosin interaction events can be observed when the dumbbell moves in one or the other direction. The binding of myosin to actin filament decouples the two beads, as seen in the fluctuation amplitudes of the bound state. The load is exerted by one side of the laser trap i.e., during the upward movement from channel 2 for event 1 and during the downward movement from channel 1. **C)** The decoupling is further illustrated with frequency distributions of displacement when the beads are free (blue line), pre-stretched in a dumbbell (yellow) or when decoupled by the myosin binding to actin (red). As expected, the fluctuations from free bead and the decoupled bead is comparable with the Full width at half maximum (FWHM) of 20.64 nm and 18.98 nm, respectively. The coupled beads through stretched actin show reduced FWHM of 14 nm. The distributions presented here for different conditions are from the same dumbbell.

Data traces acquired with mobile trap - Square wave scanning routine

Binding event detection - Manual

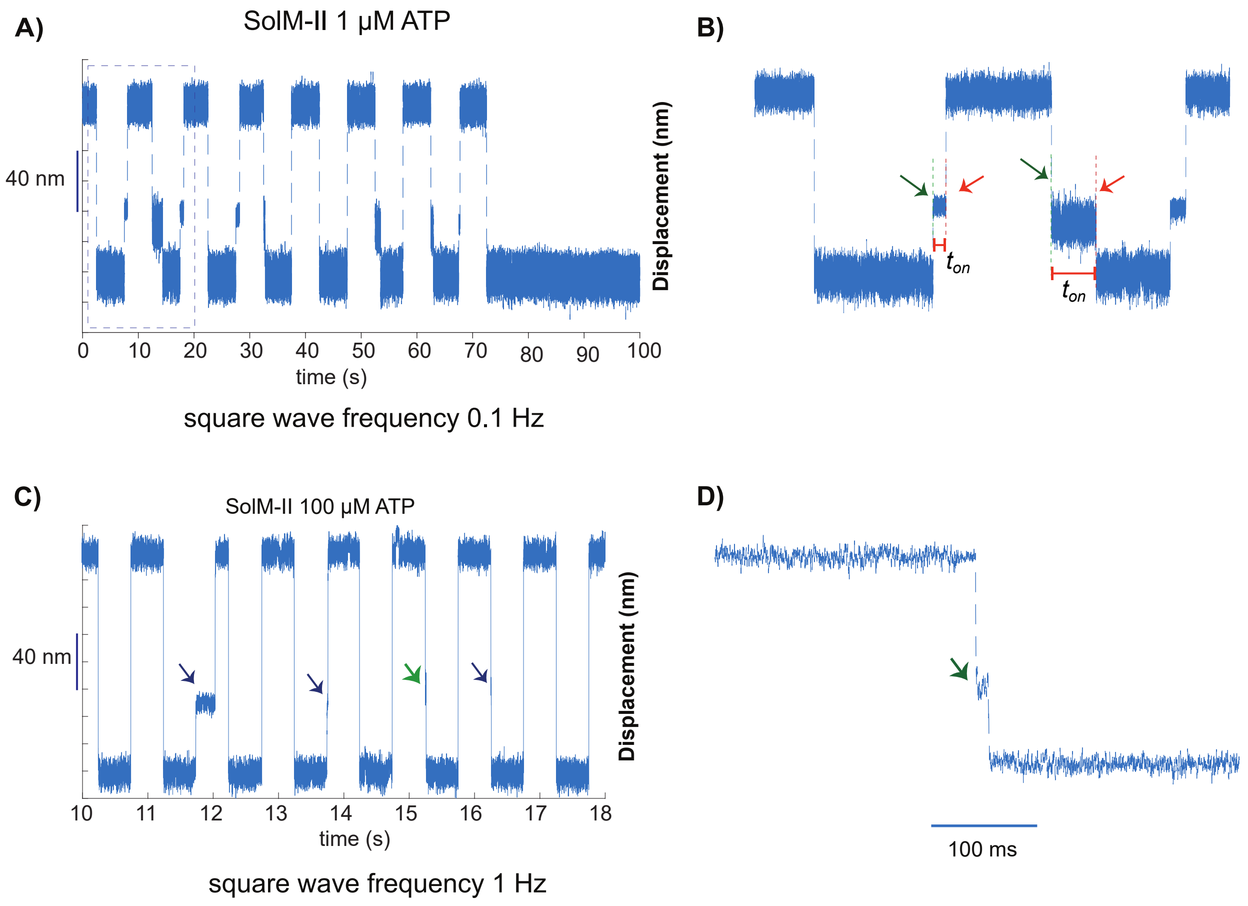

Figure S4. Event detection. **A and C)** Original data traces acquired with mobile TRAP using square wave scanning routine at 1 µM and 100 µM ATP for the SolM-II. **B and D)** the expanded view of A and C, respectively. The beginning (green arrow) and end (red arrow) of the events to mark the lifetime of the bound state 't_{on}' are shown in B. The detection of short duration events indicated with a green arrow in panel C is shown in D for clarity.

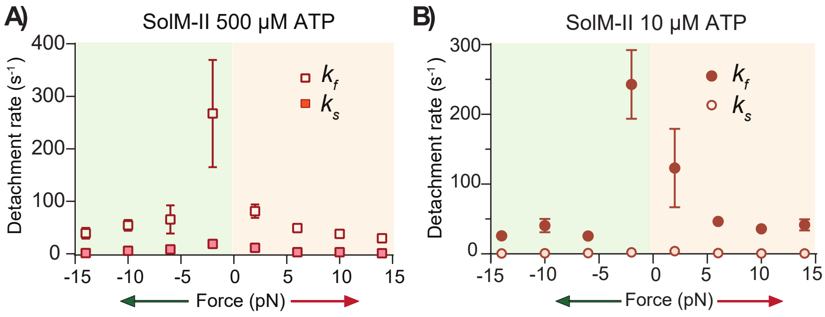

Figure S5. A and B) Force dependent detachment rates for SolM-II. A and B) Fast and slow detachments rates (k_f and k_s) at 500 µM ATP **(A) and (B)** 10 µM ATP are shown. The detachment rates estimated for different force range as described in Figure 3. Error bars- the standard error of mean (SEM) obtained from the fitting the cumulative frequency distributions. Note that for some data points, SEMs are smaller than the symbol size.
